# Twist-Induced Epithelial-to-Mesenchymal Transition Confers Specific Metabolic and Mitochondrial Alterations

**DOI:** 10.3390/cells14020080

**Published:** 2025-01-09

**Authors:** Haleigh N. Parker, Kayla L. Haberman, Tolulope Ojo, Juli Watkins, Adhwaitha Nambiar, Kayla Morales, Bernd Zechmann, Joseph H. Taube

**Affiliations:** 1 Department of Biology, Baylor University, Waco, TX 76798, USA; haleigh_svatek1@baylor.edu (H.N.P.);; 2 Center for Microscopy and Imaging, Baylor University, Waco, TX 76798, USA; 3 Dan L. Duncan Cancer Center, Baylor College of Medicine, Houston, TX 77030, USA

**Keywords:** EMT, mitochondria, TNBC, metabolism

## Abstract

Cells undergo significant epigenetic and phenotypic change during the epithelial-to-mesenchymal transition (EMT), a process observed in development, wound healing, and cancer metastasis. EMT confers several advantageous characteristics, including enhanced migration and invasion, resistance to cell death, and altered metabolism. In disease, these adaptations could be leveraged as therapeutic targets. Here, we analyze Twist-induced EMT in non-transformed HMLE cells as well as a breast cancer cell line with (MDA-MB-231) and without (MCF7) EMT features to compare differences in metabolic pathways and mitochondrial morphology. Analysis of oxidative and glycolytic metabolism reveals a general EMT-associated glycolytic metabolic phenotype accompanied by increased ATP production. Furthermore, a decrease in mitochondrial size was also associated with EMT-positive cells. However, mitochondrial elongation and spatial dynamics were not consistently altered, as HMLE Twist cells exhibit more rounded and dispersed mitochondria compared to control, while MDA-MB-231 cells exhibit more elongated and clustered mitochondria compared to MCF7 cells. These results provide further insight as to the contextual nature of EMT conferred properties.

## 1. Introduction

The ability of cells to dynamically change phenotypic characteristics in response to extracellular cues facilitates both development and homeostasis. Indeed, cells alter gene expression and the activity of signaling pathways to elicit advantageous capabilities such as enhanced migration, increased proliferation, and altered metabolism. This plasticity is crucial in maintaining human health; however, such transitions can become dysregulated in disease states [1,2,3].

One example of cellular plasticity is the epithelial–mesenchymal transition (EMT), which is implicated in development and wound healing [1,2,3]. The integrity of the epithelial layer and its apical–basal polarity are maintained by tight junctions via the homotypic interaction of the extracellular portion of E-cadherin. Upon tissue injury resulting in a breach of the epithelial layer, an initial inflammatory response prompts the expression of one or more of the EMT transcription factors: *FOXC2*, *SNAI1* (Snail), *SNAI2* (Slug), *TWIST1* (Twist), *ZEB1*, and/or *ZEB2* in epithelial cells. These transcription factors facilitate epigenetic reprogramming, activating and repressing gene expression programs, thus causing epithelial cells to lose apical–basal polarity and sever cell–cell junctions.

Within epithelial tumors, the activation of EMT facilitates initiation of metastasis and increases resistance to chemotherapy. In a growing tumor, extracellular cues can trigger *TWIST1* expression, leading to EMT and the invasion of cancer cells from the primary tumor. Such cells migrate into circulation and, through reversal of EMT, establish a secondary tumor [1,2,3,4,5,6,7,8,9]. The importance of TWIST-mediated EMT has been demonstrated in genetically engineered mouse tumor models, whereupon knocking out the gene significantly decreases metastasis [10]. Moreover, in a mouse model of squamous cell carcinoma, high expression of Twist was observed in invading cells [11].

In conjunction with changes in gene expression and cell–cell adhesion, EMT also imparts significant alterations to mitochondrial function and metabolic pathways [12,13,14,15,16,17]. Lunetti et al. found that epithelial cancer cells favor oxidative-phosphorylation (OXPHOS) metabolism, generating high amounts of ATP, while cells positive for an EMT signature upregulate glycolysis, generating less energy but also fewer damaging byproducts [18]. Intriguingly, cells in a partial EMT state (pEMT), though, are metabolically plastic and were observed by Yu et al. to employ both metabolic strategies [17]. Moreover, the epithelial, mesenchymal, and pEMT states have been further characterized by distinct metabolite abundance profiles, which also correspond to metastatic potential [19].

Here, we exploited a model of experimentally induced EMT in mammary epithelial cells (HMLE-vector vs. HMLE-Twist), driven by exogenous overexpression of TWIST1. These cells exhibit features of EMT, including increased expression of SNAI2, ZEB1, and ZEB2 and decreased expression of E-cadherin and the miR-200 family of microRNAs [20,21]. In addition, we compared two breast cancer cell lines: MCF7, a luminal ER-positive cell line that expresses E-cadherin, and MDA-MB-231, a basal-like triple-negative cell line, positive for ZEB1 but not TWIST1 expression [20]. These cells were analyzed for OXPHOS and glycolytic activity, variation in ATP production, and mitochondrial morphology and spatial dynamics. Our results demonstrate distinct differences in mitochondrial activity and morphology in the context of experimental EMT and between cancer cell lines. Specifically, we report a glycolytic metabolic phenotype and elevated ATP production both in HMLE-Twist, relative to HMLE-vector, and MDA-MB-231, relative to MCF7. We also find altered expression of transcripts of genes involved in mitochondrial fusion and fission. However, analysis of organelle morphology by TEM indicates decreased mitochondrial size in HMLE-Twist, relative to HMLE-vector, and MDA-MB-231, relative to MCF7, but inconsistent differences in elongation and spatial dynamics. These results illustrate the context-dependent nature of EMT-associated mitochondrial dynamics.

## 2. Materials and Methods

### 2.1. Cell Culture

MCF7 and MDA-MB-231 cell lines were obtained from ATCC and cultured according to provided methods using DMEM (Corning, Corning, NY, USA). HMLE and HMLE-Twist cell lines were gifted by Dr. Sendurai Mani (Brown University). Breast cancer cell lines were maintained in Dulbecco’s Modified Eagle’s Medium (DMEM) (Corning, Mediatech Inc., Manassas, VA, USA) supplemented with 10% fetal bovine serum (FBS) (Gibco, Fisher Scientific, Hampton, NH, USA) and antibiotics (Penicillin/Streptomycin) (Gibco). Immortalized human mammary epithelial (HMLE) and HMLE-Twist cells were cultured in a 1:1 ratio of Mammary Epithelium Basal Medium (MEBM) (Lonza, Walkersville, MD, USA) supplemented with Mammary Epithelial Growth Supplement (MEGS) (Gibco) and Dulbecco’s Modified Eagle Medium (DMEM)/F12 1:1 (Cytiva, HyClone, Logan, UT, USA) supplemented with Penicillin/Streptomycin (Gibco), 5 μg/mL insulin (Sigma-Aldrich Co., St. Louis, MO, USA), 10 ng/mL human epidermal growth factor (EGF) (Millipore Corp., Billerica, MA, USA), and 0.5 μg/mL hydrocortisone (Acros, Fisher Scientific, Hampton, NH, USA) as in Yang et al. [21]. All cell lines were incubated at 37 °C, 5% CO_2_ and tested bimonthly for mycoplasma.

### 2.2. Metabolic Activity Analysis

Cells were harvested in 0.15% trypsin made via a 1:1 ratio of 0.05% trypsin (Corning) and 0.25% trypsin (Gibco) then plated at 50,000 cells per well in a Seahorse XFp Cell Culture Miniplate (Agilent, Santa Clara, CA, USA) in cell media and allowed to adhere overnight. One day prior to use, a Seahorse XFp sensor cartridge was hydrated using Seahorse XF Calibrant Solution (Agilent) and placed in a non-CO_2_ incubator at 37 °C overnight. On the day of testing, compounds, provided in each kit, were suspended and diluted in Seahorse XF assay media supplemented with 1 mM pyruvate, 2 mM glutamine, and 10 mM glucose as per manufacturer instructions. Media and supplements were provided by Agilent. Prior to running each assay, cell growth media were replaced with Seahorse XF assay media with supplements. All assays were run according to manufacturer guidelines. Oxidative metabolism was evaluated using Agilent Seahorse XF Cell Mito Stress Test Kit. Glycolytic metabolism was evaluated using Agilent Seahorse XF Glycolytic Rate Assay Kit. ATP rate was evaluated using Agilent Seahorse XFp Real-Time ATP Rate Assay Kit.

### 2.3. RT-qPCR

Cells were grown, as described above, until at least 80% confluency. Cells were removed from plates and lysed using Trizol Reagent (Thermo Scientific, Waltham, MA, USA) and total RNA was extracted per the manufacturer’s guidelines. Extracted RNA was quantified using a NanoDrop Microvolume Spectrophotometer (Thermo Scientific). cDNA was generated according to manufacturer recommendations. The comparative Ct method was used for relative mRNA quantification using the formula 2^−ΔΔCt^ and Beta-Actin was used for normalization. Primers for *TWIST1*, *ZEB1*, *MFN1*, *MFN2*, *OPA1*, *MFF*, *DRP1*, and *OPA1* isoforms were obtained from Integrated DNA Technologies (Newark, NJ, USA). AzuraView GreenFast qPCR Blue Mix LR was obtained from Azura Genomics (Raynham, MA, USA). All experiments were run in technical quadruplicate and biological triplicate.

### 2.4. Transmission Electron Microscopy

Cells were grown, as described above, until at least 80% confluency. Adhered cells were washed twice with phosphate-buffered saline then fixed in 2.5% Glutaraldehyde dissolved in 0.06 M phosphate buffer (pH 7.2) for 30 min in the cell culture dish. The samples were washed in triplicate with PBS for 10 min, followed by a secondary chemical fixation for 30 min with 1% osmium tetroxide and 0.8% potassium ferrocyanide buffered with PBS (pH 7.2). Subsequently, cells were washed in triplicate with PBS for 10 min. The cells then underwent a serial dehydration in ethanol: one incubation of 50% EtOH for 10 min, one incubation of 70% EtOH for 10 min, two incubations of 90% EtOH for 10 min, and two incubations of 100% EtOH for 10 min. 

The cells were then infiltrated with a mixture of EMbed 812, epoxy resin (EMS, Hatfield, PA, USA) and ethanol through three infiltrations steps (1:2 for 1 h, 1:1 for 2 h, and 2:1 overnight) and polymerized in 100% EMbed 812 at 60 °C for 48 h. Following polymerization, samples were trimmed, sectioned 80 nm thick, and placed on a copper grid. Post-staining was completed with 1% lead citrate for 5 min and 1% uranylic acetate for 15 min. The samples were imaged using transmission electron microscopy (TEM) (ThermoFisher Spectra 300 C/TEM).

### 2.5. Post-Acquisition Image Analysis

Mitochondrial area and proximity to the nucleus and cell membrane were measured manually on TEM micro-graphs with the freehand tool in Olympus CellSens Dimension software v2.2 (Olympus America Inc., Center Valley, PA, USA). Cells were labeled prior to analysis to avoid duplication and used once for analysis. To account for the random orientation of mitochondria, a minimum of 10 different and randomly chosen cells were measured per cell type, with at least 100 mitochondria per cell line. Each mitochondrion analyzed was fully in view with minimal to no artifacts and was used once. The data collected were exported for subsequent analysis using GraphPad v10. To determine the ratio of roundness, we used the mitochondrial roundness equation from [22] (Equation (1)), where the mitochondrial length is divided by the width and subtracted from one. A value of zero represents a perfectly round mitochondrion, while an elongated mitochondrion, of varying degrees, approaches a value of one.

(1) $$Mitochondrial\;Roundness=\left|1-\frac{Wmitochondira}{Lmitochondria}\right|$$

*Wmitochondria* is the average width of the shortest diameter and *Lmitochondria* is the average length of the longest diameter of individual mitochondria measured within the cell. Proximity to the nucleus and cell membrane was determined by measuring the distance between the mitochondria to the nucleus and the cell membrane by using the closest point of mitochondria to the respective cellular component, as described above.

### 2.6. Statistical Analysis

Raw data from Seahorse Metabolic Assays were processed in Agilent Seahorse Wave Desktop software v2.6.3 (Agilent, Santa Clara, CA, USA). Raw data from qPCR were processed in Design and Analysis Software (Applied Biosystems, Thermo Scientific, Waltham, MA, USA). All processed data were analyzed in Prism v10 (GraphPad, Boston, MA, USA) using one-way ANOVAs with Tukey multiple comparison correction. Prior to ANOVA testing, TEM data were subjected to robust regression and outlier removal (ROUT, Q = 1%).

## 3. Results

### 3.1. EMT Alters Oxidative Fitness and Flexibility

**Figure 1:**
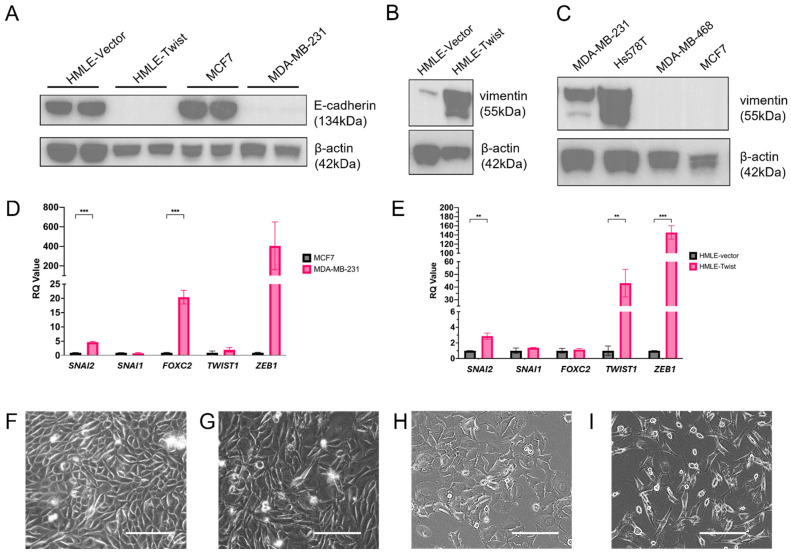
EMT status of HMLE-vector, HMLE-Twist, MCF7, and MDA-MB-231 cell lines. (**A**,**B**) E-cadherin and (**C**) vimentin expression was assayed by western blot. (**D**,**E**) Expression of *TWIST1* and *ZEB1* was assayed by qRT-PCR. (**F**–**I**) Representative images of cell morphology in (**F**) HMLE-vector, (**G**) HMLE-Twist, (**H**) MCF7, and (**I**) MDA-MB-231 cells. Scale bar represents 50 μm. Statistical analysis was performed on GraphPad Prism using one-way ANOVA significance testing; ** *p* < 0.01; *** *p* < 0.001.

Alterations to oxidative respiration have been documented in cells which have undergone an epithelial-to-mesenchymal transition (EMT). Specifically, mitochondrial oxidative phosphorylation (OXPHOS) has been observed to be decreased, while glycolysis is favored in EMT(+) cells [12,18,23,24,25,26]. To determine the capacity of Twist-induced EMT to mediate such changes, we utilized the established immortalized human mammary epithelial cells (HMLE) with a control vector (HMLE-vector) or expressing the EMT-TF TWIST1 (HMLE-Twist) [20,21]. As a means of comparison, we also included MCF7 cells, an ER-positive breast cancer cell line negative for EMT, and MDA-MB-231 cells, a triple-negative breast cancer (TNBC) cell line with EMT features [27,28]. To verify the EMT status of these cell lines, we assayed for expression of E-cadherin (Figure 1A), a marker of epithelial cells, and vimentin (Figure 1B,C), a marker of cells that have undergone EMT, by western blot as well as expression of several EMT-TFs, including *SNAI1*, *SNAI2*, *TWIST1*, and *ZEB1*, by qRT-PCR (Figure 1D,E). As expected, HMLE-vector cells and MCF7 cells express high levels of E-cadherin but not vimentin. Furthermore, HMLE-Twist cells upregulate *SNAI2, TWIST1*, and *ZEB1*, while MDA-MB-231 cells express more *FOXC2* and *SNAI2* than MCF7 but do not exhibit *TWIST1* expression. Cellular morphology is also indicative of EMT status (Figure 1F–I). Thus, while both MDA-MB-231 and HMLE-Twist cells exhibit features of EMT, MDA-MB-231 do so without enhanced *TWIST1* expression.

**Figure 2:**
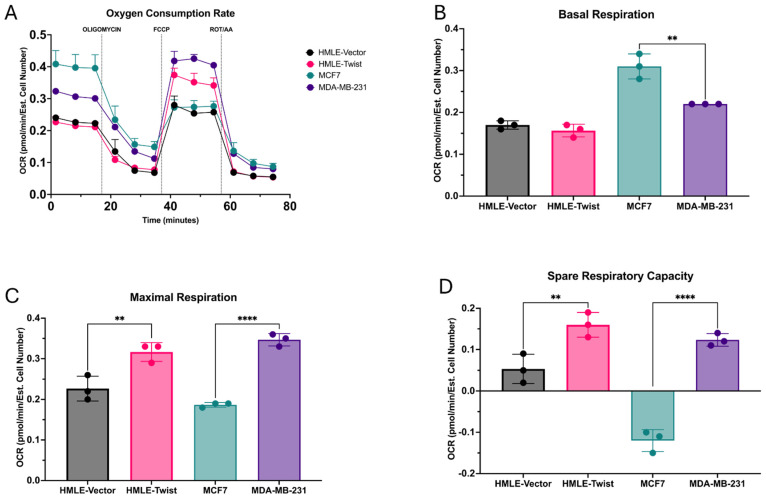
Alterations in oxidative capacity. (**A**) Oxygen consumption rate (OCR) generated via Seahorse Mitochondrial Stress Test in pmol/min/estimated cell number (ECN) was measured for the indicated cell lines. (**B**) The basal respiration before any injections and excluding non-mitochondrial respiration was measured for the indicated cell lines. (**C**) The maximal rate of respiration post FCCP injection was measured for the indicated cell lines. (**D**) The spare respiratory capacity was calculated as basal respiration subtracted from maximal respiration. Error bars denote standard deviation. Statistical analysis was performed on GraphPad Prism using one-way ANOVA significance testing (** *p* < 0.01; **** *p* < 0.0001). In all experiments, *n* = 3.

We next assayed for differences in metabolic pathway utilization by using the Seahorse Mitochondrial Stress Test. The cell lines were assayed for oxygen consumption rate (OCR) by applying a series of metabolic inhibitors and measuring the functionality of the electron transport chain (ETC) (Figure 2A). From this, several parameters describing OXPHOS activity and aerobic respiration were determined. Firstly, basal respiration, measured before the addition of any metabolic inhibitors, was found to be significantly higher in MCF7 as compared to MDA-MB-231 (Figure 2B). Following the addition of the uncoupling agent FCCP, a rapid oxidation of substrates is triggered and maximal respiration can be calculated. Interestingly, while basal respiration was found to be higher in MCF7 cells (Figure 2B), maximal respiration was significantly higher in MDA-MB-231 cells, as compared to MCF7. This result was recapitulated between HMLE and HMLE-Twist cells, with HMLE-Twist having a significantly higher maximal rate of respiration (Figure 2C). Similar results were found when spare respiratory capacity was calculated from maximal and basal respiration (Figure 2D). Indicative of oxidative fitness, the ability of the cell to respond to an energetic demand near its maximal rate of respiration, or spare respiratory capacity, was significantly higher in both EMT(+) cell lines, MDA-MB-231 and HMLE-Twist, as compared to their EMT(−) counterparts (Figure 2D).

### 3.2. EMT(+) Cells Are More Glycolytically Active than EMT(−) Cells

In addition to observations of changes to oxidative respiration in EMT(+) cells, differences in glycolysis have also been documented. Particularly, the Warburg Effect, the propensity of cells to favor aerobic glycolysis over OXPHOS, has been well recorded in association with EMT in transformed cells [2,12,13]. To expand upon this and further detail changes in glycolytic activity, we analyzed the cell lines using the Seahorse Glycolytic Rate Assay.

**Figure 3:**
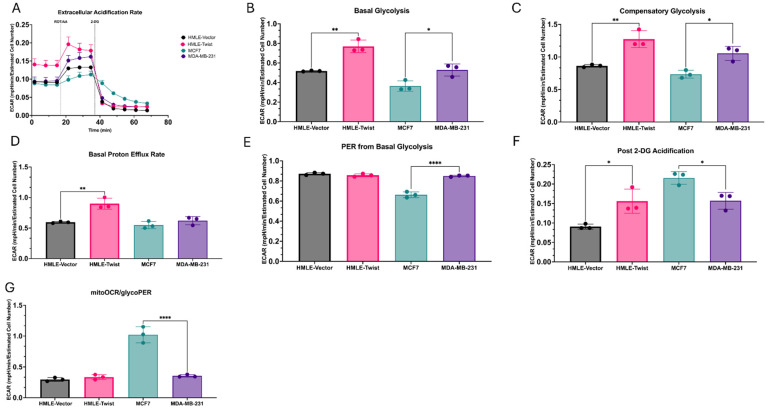
Alterations in glycolytic activity. (**A**) The extracellular acidification rate generated via Seahorse Glycolytic Rate assay in mpH/min/estimated cell number (ECN) was measured for the indicated cell lines. (**B**) The basal glycolytic rate was measured for the indicated cell lines. (**C**) The rate of compensatory glycolysis post rotenone/antimycin A injection was measured for the indicated cell lines. (**D**) The basal proton efflux rate (PER) as the number of protons effluxed into assay medium expressed as pmol/min/ECN. (**E**) The PER due to glycolysis, excluding CO_2_ dependent extracellular acidification post rotenone/antimycin A injection was calculated for the indicated cell lines. (**F**) Extracellular acidification post 2-DG and rotenone/antimycin A injection including sources of acidification other than glycolysis and mitochondrial respiration was measured for the indicated cell lines. (**G**) The ratio of mitochondrial oxygen consumption to glycolytic PER was calculated for the indicated cell lines. Error bars denote standard deviation. Statistical analysis was performed on GraphPad Prism using one-way ANOVA significance testing (* *p* < 0.05; ** *p* < 0.01; **** *p* < 0.0001). In all experiments, *n* = 3.

Cells were sequentially exposed to metabolic inhibitors and the extracellular acidification rate (ECAR) was determined (Figure 3A). ECAR is representative of the rate of glycolysis as it is generated via the efflux of protons into assay medium during the metabolism of glucose to lactate in glycolysis. From ECAR, several parameters describing glycolysis were determined. Firstly, the basal glycolytic rate before any metabolic inhibition was found to be significantly higher in both EMT(+) cell lines as compared to the EMT(−) cells (Figure 3B). Next, compensatory glycolysis, measured after the inhibition of ETC complexes 1 and 3 by rotenone and antimycin A, respectively, was also found to be higher in both MDA-MB-231 and HMLE-Twist cells (Figure 3C). This measurement determines the ability of the cells to meet energy demands utilizing primarily glycolysis.

Proton efflux into the assay medium generated from the metabolism of glucose to lactate and from the tricarboxylic acid (TCA) cycle before the addition of any metabolic inhibitors was found to be significantly different only between the immortalized cell lines, with HMLE-Twist significantly higher than HMLE-vector (Figure 3D). These results are most likely driven by protons from the TCA cycle though, as, when the proton efflux rate (PER) from glycolysis was isolated, only the cancer cell lines were found to be significantly different, with MDA-MB-231 cells having a higher glycolytic PER than the MCF7 cells (Figure 3E). This is supported by our earlier observation that MCF7 cells exhibit a significantly higher basal respiration rate (Figure 2B) and lower basal glycolytic rate (Figure 3B). These results are also supported by the significantly higher ratio of mitochondrial OCR to glycolytic PER found in the MCF7 cells as compared to the MDA-MB-231 cells (Figure 3G).

After the injection of the glycolytic inhibitor 2-DG, acidification from sources other than glycolysis and the TCA cycle can be measured. Similar to the pattern observed in cellular proton leak (Figure 3F), post 2-DG acidification was found to be higher in MCF7 cells as compared to MDA-MB-231 cells, while HMLE-Twist cells were significantly higher than the HMLE cells (Figure 3F).

### 3.3. EMT Causes an Increase in ATP Production, Driven by Glycolysis

**Figure 4:**
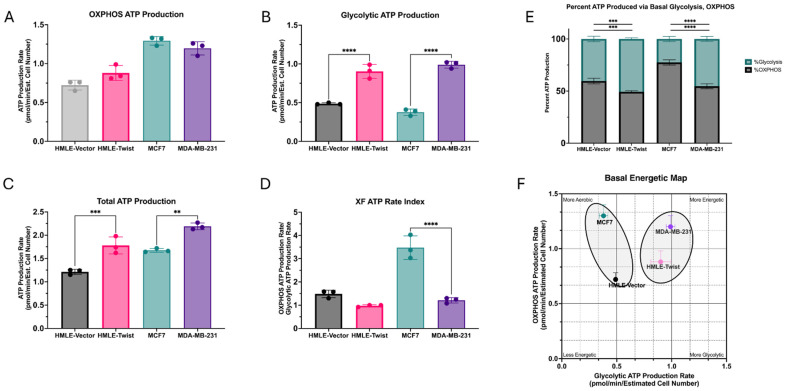
Alterations in ATP production. (**A**) The rate of ATP production via oxidative phosphorylation (OXPHOS) was measured for the indicated cell lines. (**B**) The rate of ATP production due to glycolysis was measured for the indicated cell lines. (**C**) The total rate of ATP production via OXPHOS and glycolysis was calculated for the indicated cell lines. (**D**) The XF ATP rate index as the OXPHOS ATP production rate divided by the glycolytic ATP production rate, indicative of the metabolic phenotype, was calculated for the indicated cell lines. (**E**) The percent of total ATP generated by either OXPHOS or glycolysis was calculated for the indicated cell lines. (**F**) An energetic map of basal OXPHOS and glycolytic ATP rates was calculated for the indicated cell lines. Error bars denote standard deviation. Statistical analysis was performed on GraphPad Prism using one-way ANOVA significance testing (** *p* < 0.01; *** *p* < 0.001; **** *p* < 0.0001). In all experiments, *n* = 3.

EMT(+) cells are thought to reprogram their bioenergetic pathways, supporting their enhanced proliferative and migratory capabilities [14,29,30]. Therefore, we subjected the models to the Seahorse ATP Rate Assay to better determine their metabolic profile. ATP generated from both oxidative respiration and glycolysis were determined utilizing specific metabolic inhibitors. Firstly, the ATP production rate generated from OXPHOS was found to not differ significantly between any of the cell lines (Figure 4A). However, when ATP production via glycolysis was measured, both EMT(+) cell lines, MDA-MB-231 and HMLE-Twist, were found to be significantly higher than their EMT(−) counterparts (Figure 4B). This result drives the significant differences observed in total ATP production (Figure 4C), with both EMT(+) cell lines higher than the EMT(−) cells. However, when using these values to generate the XF ATP rate index (Figure 4D), the EMT(−) MCF7 cells are found to be significantly higher than the MDA-MB-231 cells, indicative of a significantly more aerobic phenotype.

To better represent the different metabolic phenotypes generated from the assay, we plotted the oxidative ATP rate against the glycolytic ATP rate, generating a metabolic phenotype map with four quadrants: aerobic, glycolytic, more energetic, and less energetic (Figure 4F). As expected, based on results from all three metabolic assays, the EMT(+) cancer cell line MDA-MB-231 is the most energetic. While slightly less energetic, the EMT(+) non-transformed cell line HMLE-Twist is more glycolytic than oxidative, allowing both EMT(+) cell lines to be grouped in the same general metabolic phenotype. Comparatively, the EMT(−) cancer cell line, MCF7, is the most aerobic line. While also less energetic, the EMT(−) non-transformed HMLE cells are more aerobic than glycolytic, placing them within the same general metabolic phenotype as the MCF7 cells (Figure 4F).

### 3.4. EMT-Associated Changes in Mitochondrial Morphology Are Context-Dependent

Mitochondrial size and morphology are dictated by rates of fission and fusion, orchestrated by fusion proteins MFN1, MFN2, and OPA1 and fission proteins DRP1 and MFF. Significant shifts in mitochondrial morphology have been documented in EMT-positive cells; however, previous studies indicate context-specific patterns [26,31,32,33,34,35,36]. Thus, we quantified mitochondrial metrics using images taken by transmission electron microscopy (TEM) and correlated this analysis with expression of fission and fusion proteins to identify morphological changes and potential regulators in the context of our models.

**Figure 5:**
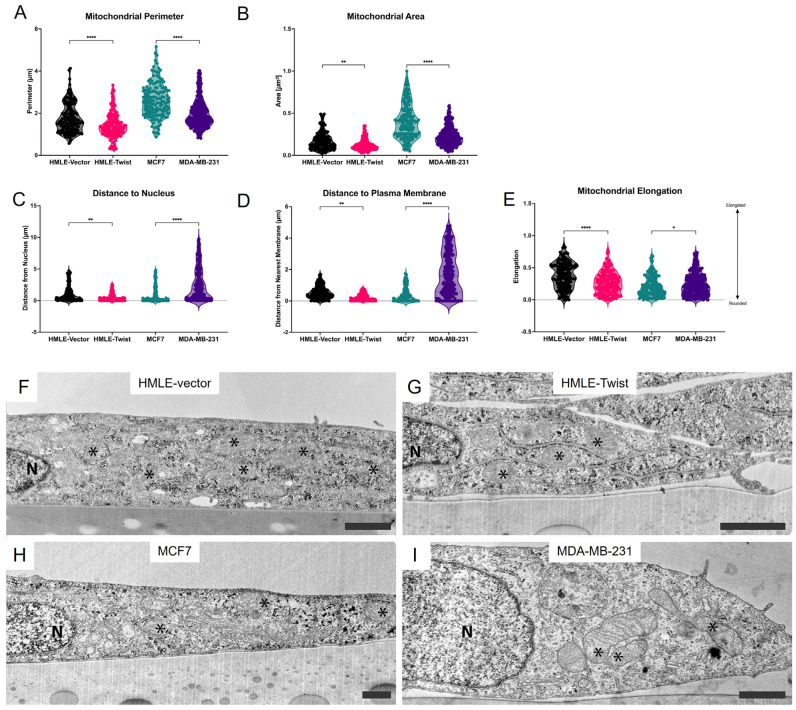
Alterations in mitochondrial morphology. (**A**) Perimeter and (**B**) area of individual mitochondria were measured by analysis of TEM images. (**C**) Distance of mitochondria from the nucleus and (**D**) nearest cell membrane was measured by analysis of TEM images. (**E**) Mitochondrial elongation quantified as in [22], whereupon a value of zero represents a perfectly round mitochondrion, while an elongated mitochondrion, of varying degrees, approaches a value of one. (**F**–**I**) Representative TEM images of indicated cell lines. Scale bar represents 1 μm. Mitochondria are denoted by ‘*’. Statistical analysis was performed on GraphPad Prism using ROUT outlier removal (Q = 1%) and one-way ANOVA significance testing (* *p* < 0.05; ** *p* < 0.01; **** *p* < 0.0001). In (**A**–**E**), *n* ≥ 12 cells, or 162 mitochondria.

Analysis of TEM images reveals elevated mitochondrial perimeter and area in both EMT(−) cell lines (Figure 5A,B). This agrees with studies reporting higher rates of mitochondrial fusion in epithelial cell lines and greater fragmentation in mesenchymal cells [26,35,36]. Next, we sought to determine mitochondrial localization within each cell line. Noted by measuring the shortest distance between each mitochondrion and the nucleus, MDA-MB-231 mitochondria are significantly further from the nucleus as compared to MCF7 cells (Figure 5C). Contrastingly, mitochondria in HMLE-vector cells are a greater distance from the nucleus than mitochondria in HMLE-Twist cells (Figure 5C). This same pattern is observed when measuring the shortest distance between the mitochondria and the plasma membrane (Figure 5D). These data sets, when combined, indicate in both MDA-MB-231 and HMLE cells that mitochondria tend to cluster in the cytoplasm, while, in HMLE-Twist and MCF7 cells, mitochondria are more dispersed. The dispersion in the Twist-induced EMT model is consistent with data in MCF10A cells also induced to express Twist, which exhibit more dispersed, fragmented mitochondrial networks [26]. Conflicting with this model, though, is the significant decrease in elongation in HMLE-Twist cells compared to HMLE-vector cells (Figure 5E), as previous reports observed little change in mitochondrial length. Also, in disagreement with other reports, mitochondria in MDA-MB-231 are slightly more elongated than those in MCF7 cells (Figure 5E) [35]. Representative images are provided in Figure 5F–I. Together, these imaging data indicate that mitochondrial size is consistently altered in EMT(+) cells, regardless of cancer status, but changes in mitochondrial localization and shape are context-dependent.

**Figure 6:**
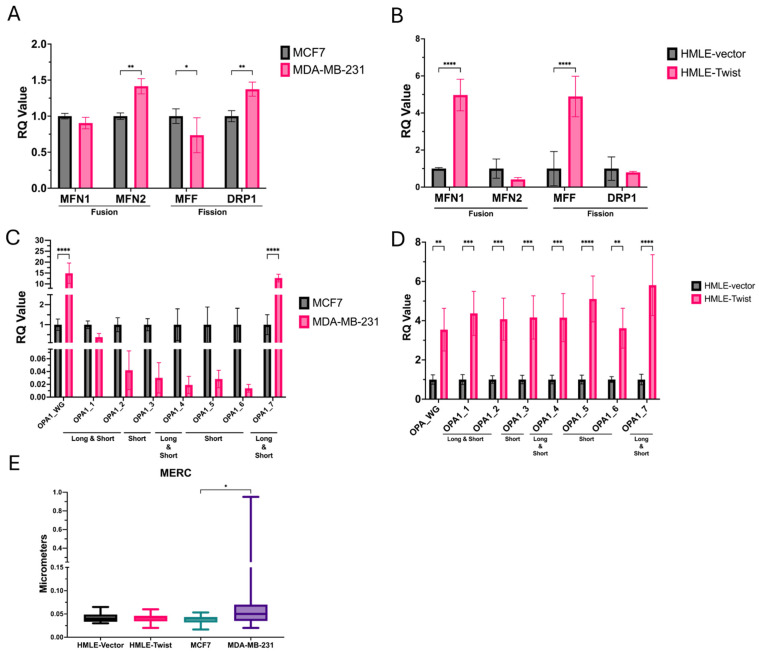
Alterations in expression of genes regulating mitochondrial fusion and fission. (**A**–**D**) qPCR analysis of mitochondrial-dynamics-related transcripts. Error bars denote standard deviation. *n* = 3. (**E**) Contact between individual mitochondria and the ER was determined via analysis of TEM images. *n* ≥ 12 cells, or 162 mitochondria. Statistical analysis was performed on GraphPad Prism using ROUT outlier removal (Q = 1%) and one-way ANOVA significance testing (* *p* < 0.05; ** *p* < 0.01; *** *p* < 0.001; **** *p* < 0.0001).

To corroborate observations from TEM imaging analysis, we analyzed mRNA expression for mitochondrial fission and fusion regulators *MFN1*, *MFN2*, *MFF*, *DRP*, and *OPA1* (Figure 6A–D). In the cancer cell lines, expression of both *MFN2* and *DRP1* is significantly increased, while *MFF* expression is significantly lower in MDA-MB-231 cells compared to MCF7 cells (Figure 6A). In HMLE-Twist cells, expression of *MFN1* and *MFF* is significantly increased over HMLE-vector (Figure 6B). *OPA1* is expressed as several different isoforms (1–7) due to alternative splicing of exons 4, 4b, and 5b [37]. Spliced isoforms can encode for either or both long or short protein isoforms due to the inclusion or exclusion of protease cleavage sites. Shorter isoforms are more associated with mitochondrial fission, while longer isoforms are associated with mitochondrial fusion. Importantly, regulation of apoptosis, cristae structure, fusion, and energetics is dependent on the presence of both short- and long-form isoforms. In both models of EMT, transcriptional expression of *OPA1* is increased in the EMT(+) cell lines (Figure 6C,D). However, in MDA-MB-231 cells, this difference appears to be driven by higher expression of isoform 7 (Figure 6C), which encodes both short and long protein isoforms, while other isoforms are less abundant in MDA-MB-231 when compared to MCF7 [38]. Contrastingly, expression of all *OPA1* isoforms is significantly increased in Twist-induced EMT (Figure 6D). Whether *OPA1* expression is regulated primarily by splicing in MDA-MB-231 cells remains to be determined. Finally, as *MFN2* is involved in mitochondrial tethering to organelles, in particular, the endoplasmic reticulum (ER), we measured the contact between the two organelles [34]. In agreement with the transcriptional difference in *MFN2*, we observed significantly more mitochondrial–ER contact in MDA-MB-231 cells than MCF7 cells (Figure 6E). While this aligns with some previous studies reporting elevated DRP1 expression in MDA-MB-231 cells, the differences between the two models of EMT here support the context-dependent nature of mitochondrial dynamics [32,33].

## 4. Discussion

Multiple mechanisms of cellular plasticity, of which EMT is but one example, are required to facilitate development and maintain human health [2,5,12,14]. Here, we delineate mitochondrial and metabolic alterations within a model of Twist-induced EMT in mammary epithelial cells as compared to two breast cancer cell lines with and without EMT features.

While basal oxidative respiration differs only between the two breast cancer cell lines, oxidative fitness and metabolic flexibility were significantly elevated in both MDA-MB-231 vs. MCF7 and HMLE-Twist, vs. HMLE-vector, arguing that this feature is not directly tied to TWIST1 expression but to EMT more generally. A higher degree of metabolic flexibility is potentially useful to a cell undergoing EMT-driven metastasis or wound healing, during which sudden demands in energy frequently occur [2,13,14]. The rapid oxidation of substrates, which elevates oxidative metabolism, provides cells with sufficient ATP to fuel migration [17,30,39]. As both cancer and non-cancer cells with EMT exhibit increased migratory capacities, elevated oxidative flexibility may be a commonly conferred characteristic, regardless of TWIST1 expression.

EMT(+) cells were also found to have significantly higher rates of both basal and compensatory glycolysis, providing further support for EMT-conferred metabolic plasticity. Compensatory glycolysis measures the ability of cells to meet energy demands utilizing solely glycolysis, providing insight as to cell dependency on oxidative metabolism [40]. The elevated capacity to depend on the glycolytic pathway observed in EMT(+) cells may provide an advantage under hypoxic or nutrient-deprived conditions often found in metastasis and wound healing [30,41,42]. Furthermore, the ability to utilize glycolysis rather than oxidative metabolism can reduce the production of cell-damaging byproducts, such as reactive oxygen species (ROS) [17,43].

During the breakdown of glucose to lactate in glycolysis, and as an end product of the TCA cycle, protons are effluxed from the cell. The proton efflux rate (PER) is used to determine the degree of pathway activity. PER associated with glycolytic efflux is termed GlycoPER, while PER associated with the TCA cycle is MitoPER [40]. Here, we found basal PER, the sum of GlycoPER and MitoPER, to be elevated in HMLE-Twist vs. HMLE-vector, while GlycoPER was elevated in MDA-MB-231 vs. MCF7. Taken together, this indicates an elevated MitoPER in Twist-induced EMT and a higher degree of activity in the TCA cycle. However, comparing the two cancer cell lines, the minor difference in basal PER implies a higher MitoPER in the EMT(−) cell line. This aligns with MCF7 exhibiting an elevated basal respiration and higher mitoOCR/GlycoPER ratio relative to MDA-MB-231. As MitoPER is a measure of the activity of the TCA cycle, the elevated activity observed in the HMLE-Twist cells compared to HMLE-vector but not in other measures of respiration may be due to other pathways with downstream effects on the TCA cycle, such as fatty acid metabolism [44,45]. This is substantiated by the significant differences observed after the addition of 2-DG, a glycolysis inhibitor, to measure proton efflux emitted from sources other than the TCA cycle and glycolysis [40]. Both HMLE-Twist and MCF7 cell lines may be utilizing alternative sources of TCA cycle components not observed in the MDA-MB-231 cell line. This reveals a previously unknown characteristic of EMT in cancer not induced via TWIST1.

Ultimately, the end product of cellular metabolism is the generation of ATP, used for nearly all cellular processes. While little difference was found between cell lines for ATP derived from oxidative metabolism, more ATP was generated by glycolysis in EMT(+) cells, driving a significantly higher total production of ATP. High ATP production corroborates previous results in which EMT(+) cells are more metabolically active and plastic [15,23,29]. The ATP rates from both oxidative metabolism and glycolysis can be used to generate energetic phenotypes. Here, we show that both EMT(−) cell lines are in an aerobic phenotype, with the non-cancer cell line being less energetic/metabolically active, while the EMT(+) cell lines are in a glycolytic, energetic metabolic phenotype (Figure 4F).

Mitochondria undergo changes to their morphology via fission and fusion proteins, impacting their capacity to support OXPHOS and generate ATP. The fission proteins, MFF and DRP1, work to split mitochondria, generating more organelles that are generally smaller in size and a mitochondrial network that is more fragmentated. Fusion proteins, MFN1/2 and OPA1, fuse the inner and outer membranes of mitochondria, producing larger organelles [26,31,32,33,34,35]. Using transmission electron microscopy (TEM), we analyzed the morphology of individual mitochondria in multiple cells from each model. In both models, EMT(−) cells exhibit larger mitochondria but, when measuring the expression of fusion and fission mRNA, the HMLE-Twist cells exhibited elevated transcription of *MFN1* and *MFF* (Figure 6B), while MDA-MB-231 cells express more *MFN2* and *DRP1* but less *MFF* than MCF7 cells. Additionally, in both models, *OPA1* transcriptional expression is elevated in EMT(+) cells (Figure 6C,D). While this increased expression appears to be driven by isoform 7 and somewhat attenuated by decreased expression of isoforms 1–6, in MDA-MB-231 cells, expression of all isoforms is significantly elevated in HMLE-Twist cells. Therefore, OPA1 expression is transcriptionally elevated in both models of EMT but likely driven by different isoforms. On the other hand, expression of fusion genes, *MFN1* and *MFN2*, is not directly tied to EMT status. Lastly, in agreement with elevated transcription of *MFN2*, a greater amount of mitochondrial contact with the ER was observed in MDA-MB-231 cells, compared to MCF7, indicating a possible increase in ER stress [46].

In sum, changes in mitochondrial morphology are distinct when comparing Twist-induced EMT in HMLE cells to a model of EMT lacking Twist expression (MDA-MB-231), while alterations in metabolic activity are shared. It is also likely that genetic changes necessary for the onset of cancer, in addition to EMT, influence these morphological changes, rather than EMT alone.

The correlation between EMT-associated metabolic states and alterations in mitochondrial morphology requires further investigation, as many studies have yielded disparate results. In epithelial cells, larger and more tubular mitochondria have been observed. This phenotype often correlates with higher rates of mitochondrial fusion, an oxidative phenotype, and expression of mitochondrial fusion proteins [26,32,35]. Contrastingly, mesenchymal cell types generally possess smaller, more fragmented mitochondria with higher expression of mitochondrial fission proteins [26,32,35,36]. In the context of EMT, though, mitochondrial dynamics seem to be significantly context-dependent. For example, inducing EMT via Twist in MCF10A cells resulted in high expression of DRP1 and a fragmented mitochondrial network [26,35,36]. However, Twist-induced EMT in the mammary mouse line NMuMG resulted in increased mitochondrial fusion [34]. Similarly, EMT induction via TGFβ in MCF12A cells or via the transcription factor Snail in HMECs also lead to observations of fused mitochondria [34]. Other studies have examined the effects on EMT by dysregulation of fission and fusion proteins. In MCF7 cells with increased expression of fission protein MFF, decreases in oxygen consumption were accompanied by decreases in EMT-related protein expression [33]. TCGA data indicate higher expression of DRP1 in patients alive 5 years post diagnosis [31]. These findings, however, do not agree with other reports in which downregulation of DRP1 leads to decreased invasiveness of cancer cells [35]. The complexity of mitochondrial dynamics in the context of EMT necessitates further examination and clarity so metabolically targeted therapies may be improved. 

## Data Availability

Data available upon reasonable request.
